# Topological edge state cavities: simultaneous enhancement of quality factor and free spectral range

**DOI:** 10.1038/s41377-025-02104-5

**Published:** 2026-01-02

**Authors:** Shaoqi Ding, Zhihao Wang, Cuicui Lu

**Affiliations:** Key Laboratory of Advanced Optoelectronic Quantum Architecture and Measurements of the Ministry of Education, Beijing Key Laboratory of Nanophotonics and Ultrafine Optoelectronic Systems, State Key Laboratory of Chips and Systems for Advanced Light Field Display, Center for Interdisciplinary Science of Optical Quantum and NEMS Integration, School of Physics, Beijing Institute of Technology, Beijing, 100081 China

**Keywords:** Photonic crystals, Nanocavities

## Abstract

A novel topological edge state cavity has been realized to enhance the quality factor and free-spectral range, simultaneously, which opens avenues for developing robust high-performance photonic integrated devices.

Photonic micro-cavities serve as fundamental building blocks for a variety of applications in photonic integrated circuits^1,2^. One of the key features of photonic micro-cavities is the finesse, which is important for achieving strong light confinement and long light–matter interaction times. Finesse, defined as the ratio of the free spectral range (FSR) to the resonance linewidth, necessitates the simultaneous enhancement of both the *Q* factor and FSR for achieving strong light confinement and prolonged light–matter interaction times. One method to improve the *Q* factor of the cavities is to increase their round-trip length; meanwhile, this inherently decreases the FSR, since FSR is inversely proportional to the cavities’ round-trip length. In recent years, topological valley photonics has introduced a new scheme for light manipulation in a robust way^3,4^. Now, writing in Light: Science & Applications, Ranjan and his colleagues report a topological edge state cavity (TESC) that simultaneously enhances both *Q* factor and FSR to improve the performance of photonic micro-cavities^5^.

In general, most of the conventional integrated photonic cavities fall into three categories: FP cavity, whispering-gallery mode cavities, and photonic crystal (PC) cavities^1^. Although enlarging the round-trip length enables these cavities to achieve a high *Q* factor, the concomitant reduction in FSR imposes an intrinsic trade-off that ultimately constrains their finesse. In recent years, topological photonics has provided a new platform to manipulate light that has attracted enormous interests^6,7^. With the help of topological protection, the topological edge modes can transmit without back-reflection even in the presence of sharp bends and defects. Topological valley photonics not only have topological edge modes, but also easily designed with all-dielectric materials, since it only needs inversion symmetry breaking. While early works demonstrated that bending the topological interface into a closed loop could create a topological cavity^8,9^. However, how to design the cavities to enhance both the *Q* factor and the FSR simultaneously still remains a challenge.

Ranjan and his colleagues propose a new mechanism that can both enhance *Q* factor and FSR of cavities, simultaneously^5^. They construct the TESC from a closed-path topological interface and tune the inversion symmetry broken strength of the valley photonic crystal (VPC) instead of changing the cavity size. Specifically, each unit cell of VPC consists of two triangular air holes with side length ${L}_{1}=(a+\Delta L)/2$ and ${L}_{2}=(a-\Delta L)/2$, where $\Delta L$ indicates the inversion symmetry broken strength (Fig. 1a). By controlling the difference in size length $\Delta L$, they could modify the properties of the topological edge state. As $\Delta L$ decreased, the topological edge state is shifted from above to below the light line (Fig. 1b), which suppresses radiation leakage and dramatically enhances the intrinsic *Q* factor of TESC modes by three orders. Concurrently, the decrease can modify the dispersion of topological edge state; its dispersion slope becomes steeper, leading to a larger group velocity and a reduced effective refractive index ${n}_{g}$, thereby further enhancing the FSR (Fig. 1c). This approach successfully realizes a high-finesse topological cavity.

**Fig. 1:**
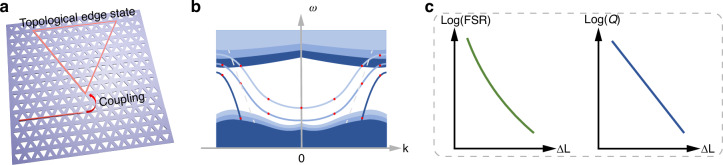
**Topological edge state cavities**. **a** TESC coupled with a topological waveguide. The unit cell of the valley photonic crystal (VPC) consists of two equilateral air holes with side lengths ${L}_{1}$ and ${L}_{2}$. **b** Edge states in VPCs. Waves are confined and guided through topological edge states in TESC. **c** Evolution of FSR and *Q* with *R* and Δ*L* of TESCs. Reducing $\Delta L$ can effectively simultaneously increase FSR and *Q*

Furthermore, the development of topological edge states can be extended to nonlinear regimes, thereby revealing richer physical phenomena. In a recent paper of Light: Science & Applications, Jie Ma and colleagues report the first experimental observation of the formation of nonlinear edge states in the density population evolution and participation ratio with increasing interaction^10^. They construct the trimer array in momentum space and employ Feshbach resonance to tune the nonlinear strength. By implementing three distinct initialization schemes, they find that as the nonlinear strength increases, the ultracold atomic gases become more localized at the boundaries of the lattice. This work opens a new avenue for exploring nonlinear topological physics in ultracold atomic systems.

The mechanism of nonlinearity-induced localization brings us inspiration for enhancing cavity performance. While the TESC already confines the light, its *Q* factor is still limited by the leakage of the edge mode into the bulk region of the PC. We can introduce optical nonlinearity into the cavity to induce the self-trapping effect for photons. This method would force the light to be more localized within the topological interface and further enhance the *Q* factor.

Looking ahead, topological photonic cavities, enabled by topological protection, achieve robust light confinement and transport with inherent resistance to defects, low losses, and high stability, offering a novel platform for photonic integrated circuits, nonlinear optics, and quantum information processing. Furthermore, topological photonic cavities exhibit promising potential in applications such as single-photon sources, high-sensitivity sensing, and frequency filtering. Especially, the topological cavities provide a platform to realize high-performance nano-lasers^11,12^. For on-chip integration, improving compatibility with established platforms such as silicon photonics and silicon nitride will be essential to realize large-scale topological photonic networks. In the field of nonlinear optics, strong light confinement can be exploited to enhance nonlinear effects, enabling low-power frequency conversion, soliton generation, and coherent state manipulation. In quantum optics, the topological protection mechanism may facilitate the preparation of robust quantum states, resilient entanglement distribution, and even the exploration of topological qubits, paving the way for fault-tolerant quantum computing and quantum communication. Beyond these domains, combining topological photonics with concepts from acoustics, superconductivity, and other disciplines could further inspire new physical mechanisms and device architectures.
